# Specific training for LESS surgery results from a prospective study in the animal model

**DOI:** 10.1590/S1677-5538.IBJU.2014.0658

**Published:** 2016

**Authors:** Giovannni Scala Marchini, Italo D. Fioravanti Júniori, Leonardo V. Horta, Fabio C. M. Torricelli, Anuar Ibrahim Mitre, Marco Antonio Arap

**Affiliations:** 1 Instituto de Ensino e Pesquisa do Hospital Sírio Libanês, São Paulo, Brasil;; 2 Hospital das Clínicas da Universidade de São Paulo Faculdade de Medicina de São Paulo, Brasil

**Keywords:** Surgical Procedures, Operative, Laparoscopy, Disease, Hand-Assisted Laparoscopy

## Abstract

**Objective:**

to prospectively evaluate the ability of post-graduate students enrolled in a laparoscopy program of the Institute for Teaching and Research to complete single port total nephrectomies.

**Materials and Methods:**

15 post-graduate students were enrolled in the study, which was performed using the SILS^tm^ port system for single-port procedures. All participants were already proficient in total nephrectomies in animal models and performed a left followed by a right nephrectomy. Analyzed data comprised incision size, complications, and the time taken to complete each part of the procedure. Statistical significance was set at p<0.05.

**Results:**

All students successfully finished the procedure using the single-port system. A total of 30 nephrectomies were analyzed. Mean incision size was 3.61 cm, mean time to trocar insertion was 9.61 min and to dissect the renal hilum was 25.3 min. Mean time to dissect the kidney was 5.18 min and to complete the whole procedure was 39.4 min. Total renal hilum and operative time was 45.8% (p<0.001) and 38% (p=0.001) faster in the second procedure, respectively. Complications included 3 renal vein lesions, 2 kidney lacerations and 1 lesion of a lumbar artery. All were immediately identified and corrected laparoscopically through the single-port system, except for one renal vein lesion, which required the introduction an auxiliary laparoscopic port.

**Conclusion:**

Laparoscopic single-port nephrectomy in the experimental animal model is a feasible but relatively difficult procedure for those with intermediate laparoscopic experience. Intraoperative complications might be successfully treated with the single-port system. Training aids reducing surgical time and improves outcomes.

## INTRODUCTION

Over the last two decades, laparoscopy has revolutionized urological practice. Several series have reported promising results for simple (1) and complex upper tract procedures involving benign (2) and malignant diseases (3).

Laparoendoscopic single-site surgery (LESS) represents the latest innovation in laparoscopic surgery. It aims to minimize postoperative pain and time to complete recovery with improved cosmesis. However, LESS is known to be a challenging procedure since triangulation, a basic principle of laparoscopic surgery, is lost. Therefore, instruments often collide and the procedures are usually associated with poor surgeon and assistant ergonomics (4).

Similarly to a standard laparoscopic surgery, LESS has a learning curve and requires training in technical skills and spatial awareness, as these are different from skills required for open surgery or standard laparoscopy. The aim of the present study was to prospectively evaluate the feasibility and morbidity of single-port nephrectomy performed by post-graduate students in live animal models.

## MATERIALS AND METHODS

### Participants

After having undergone an extended training in urologic experimental laparoscopic surgeries in the animal model at the accredited center of the Institute for Teaching and Research of our Institution, 15 graduated urologists of the post-graduation laparoscopy Urology course were invited to participate in the study. The program comprises a year-long post-graduate course in which students spend three full days per month (one module of a total of ten modules) learning urologic laparoscopic principles and skills. As part of their training, they spend 12 hours per module practicing surgical skills and procedures in live animal models. All invited students were in the two final modules of the annual course and were proficient in laparoscopic total nephrectomies performed in the porcine model. All students accepted to join in the study and were considered suitable.

### Single-Port System and Nephrectomy in the Animal Model

The experimental procedures in the wet laboratory consisted in the evaluation of basic nephrectomy tasks (port placement, renal hilum control, and renal dissection) in a porcine model using Single-Incision Laparoscopic Surgery SILS^TM^ (Covidien, Norwalk, CT) port system. The SILS port is an FDA approved, single-incision flexible device, which may be inserted via an open technique through a skin and fascial incision as small as 15mm. It allows access for three 5mm cannulas or one 12mm cannula and two 5mm cannulas. The students had no previous experience with any single-port system and were allowed 15 minutes to familiarize with the instruments immediately before the initiation of the procedure.

Fifteen swine (mini pig BR) weighting 30-35Kg were used in the study. All animals were acquired from the same facility. The protocol was approved by the ethical committee of our institution. In all animals, anesthesia was induced with a combination of intramuscular ketamine (5mg/Kg) and midazolam (0.5mg/Kg) and maintained with continuous intravenous propofol (8mg/Kg) and inhalatory isoflurane (2%) infusions. All procedures were performed with the animal in the flank position. After the first nephrectomy, the incision site was closed and the animal repositioned to the contralateral procedure. A new incision site was used 1cm above or beyond the first one. At the end of the procedures, all pigs were euthanized. All animals were intubated and ventilated and the abdomen was placed on the edge of the bed to prevent instrument collision and mobility limitation.

All students performed exactly the same surgical tasks: a vertical trans-umbilical incision was made and students were oriented to do the smallest incision for trocar placement. The rectal fascia was then identified and opened under direct visualization. Two holding stitches were placed on either side of the fascia to facilitate easier port placement. After abdominal insufflation, a 30º 10mm laparoscope was placed. Renal dissection was performed using a 23cm long grasper, a 23cm-long scissor and the Ethicon Harmonic Scalpel^TM^ (Cincinnati, OH, USA). The peritoneum was incised and the renal hilum was identified. The artery and veins were manually taken separately using 4-0 silk knots. After complete control of the renal hilum, the kidney was completely mobilized using instrument dissection and harmonic energy.

The following parameters were evaluated for each student: incision size (cm), time to insert the SILS Port^®^, time to mobilize and divide the renal pedicle, time to dissect and mobilize the kidney, and total surgery time. Each student performed a left followed by a right nephrectomy. The same parameters were compared between the first and second procedures to evaluate the learning curve effect. We also evaluated intraoperative complications and the ability to treat them in case they occurred. Each student performed one right and one left total nephrectomy. After the procedures, students were individually questioned about the two most important technical challenges of single-port.

### Statistical analysis

Statistical analysis was performed using SPSS^TM^ version 20 (SPSS, Inc., Chicago, IL, USA). Results were described as mean, standard deviation and range values. Paired T Test was used to compare parameters of first and second procedure for each student. Statistical significance was set at two-tailed p<0.05.

## RESULTS

A total of 30 nephrectomies were evaluated. Nephrectomies were successfully completed by all students. Intraoperative data is detailed in Table-1. Mean incision size was 3.61±0.8 (2.5-5) cm. Mean time for trocar insertion was 9.6±3.4 (3-17.4) min. Mean time to dissect and control the renal hilum was 25.3±10.4 (7.9-43) min and to dissect the kidney was 5.2±1.5 (2.1-9.5) min. Finally, mean time to complete the whole procedure was 39.4±12.3 (20.5-59.3) min.

**Table 1:** Single-port nephrectomy task and performance evaluation.

Tasks Performance	Mean±SD	range
Incision size (cm)	3.61±0.8	2.5-5.0
Time to trocar insertion (min)	9.61±3.4	3.0-17.4
Time to dissect renal hilum (min)	25.3±10.4	7.9-43.0
Time to kidney dissection (min)	5.18±1.5	2.1-9.5
Total procedure time (min)	39.4±12.3	20.5-59.3

Procedure Feasibility and Morbidity	N	%

Successful procedures	30	100%
Complications	6	20%
Conversion to standard laparoscopy	1	3.4%

**Figure 1:**
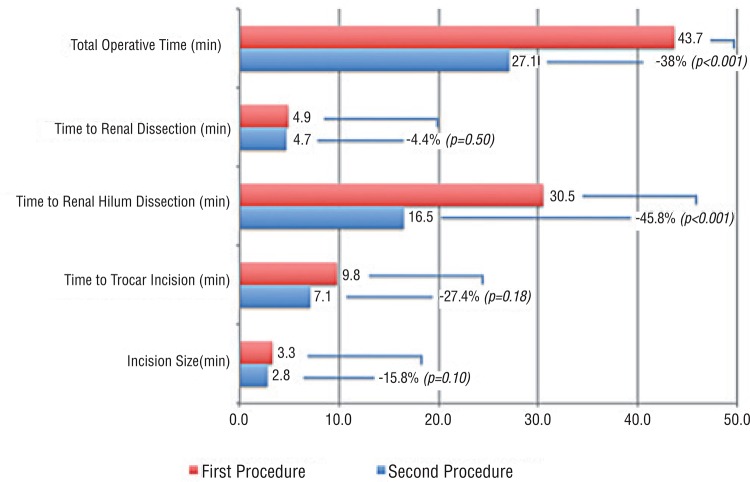
Individual task comparison between the first and second SILS nephrectomy.

Mean incision size was 0.5cm (15%) shorter in the second procedure, although not statistically significant (p=0.10) (Figure-1). Mean time to trocar insertion was 27.4% (2.7 min) faster in the second nephrectomy (p=0.18). Although time to dissect renal hilum was faster (45.8%; 13.9 min; p<0.001), time to dissect the kidney was very similar (0.2 min faster in the second surgery; p=0.5). Total operative time was significantly shorter (16.6 min; 38%) in the second nephrectomy (p=0.001).

Intraoperative complications were seen in 6 (20%) procedures (Table-1): 3 renal vein lesions, 2 small kidney lacerations, and 1 lesion of a lumbar artery. All complications were immediately identified and lesions were corrected using single-port instruments, except for one renal vein lesion, which required the introduction of an auxiliary 5mm laparoscopic port (3.4% conversion rate). Blood loss was minimal during the procedures and could not be quantified.

Instrument collision and spatial awareness were the most common time consuming problems found by the surgeons. Mobilization of the camera was often related as difficult by the assistants due to the narrow operative field and the small spatial mobilization, occasionally preventing adequate exposure of the operative field.

## DISCUSSION

Standard laparoscopic nephrectomy requires fine surgical skills since renal pedicle mobilization may be difficult and vascular complications are usually severe and life threatening. In addition, it requires the introduction of at least 3 ports and each trocar inserted increases the risk of bleeding, internal organ injury, and port-site hernia, also compromising cosmetic results. Single-port access has been developed in order to reduce those complications and with the benefits of less postoperative pain, faster convalescence and better cosmetic results (5). Nevertheless, single-port surgery is known to be a challenging technique, as maneuverability is poor inside and outside the abdomen, there is no triangulation of the conventional laparoscopic instruments, and collision between the instruments and the camera is frequent (6). In order to overcome such limitations, new flexible instruments were developed and intensive training is required to achieve results similar to those of standard laparoscopic technique.

In this study, we evaluated the ability of post-graduate students to complete basic laparoscopic nephrectomy skills using a SILS Port®. All nephrectomies were completed through single-port access and one renal vein lesion required the introduction of an auxiliary 5mm port to control a small bleeding. Our results show that single port nephrectomy is feasible, even for surgeons with no previous experience with single-port devices. Usually pedicle ligation is achieved with clip ligation of the vessels. However, we decided to use manual knots for such task, in order to better evaluate the procedure in case clip ligation was unavailable or unsuccessful. Despite the known difficulty during pedicle ligation with manual knots, all students successfully completed this task. Although we analyzed the learning curve effect with few cases, we found significant differences from the first to the second procedure. This highlights the importance of training the exact procedure with the correct materials for laparoscopy, leading to faster procedures parallel to better outcomes.

Our results revealed a great variability between students in all steps of the procedure. This was expected and consistent with other early series of laparoscopic and single-port nephrectomies in which the learning curve plays an important role in the duration of the procedure (7). Instrument collision and spatial awareness were the most common time consuming problems found by the surgeons. In addition, according to the assistant report, mobilization of the camera was often difficult since the operative field was narrow and a small spatial mobilization usually prevented ideal visualization of the operative field. Autorino et al. have already compared mini-laparoscopy, laparo-endoscopic single-site surgery and natural orifice transluminal endoscopic surgery for total nephrectomy (8). They found no differences in overall operating time, or time to dissect and manage the renal vascular hilum, however time to gain access was faster with the single-site technique. The subjective perception of the degree of difficulty trended in favour of mini-laparoscopy, but no significant difference was found in regards of surgeon’s impression as compared with their expectations.

Single-port access has been introduced as a method that could potentially reduce standard laparoscopic complications, e.g. internal organ and vascular injury, as it does not require needle or blind port placement and there is no need for extra ports (6). Nevertheless, current data shows no benefit of single-port over standard laparoscopy in terms of operative time, blood loss or complication rates (9, 10). This is probably due to a longer learning curve in acquiring specific skills using single-port access compared to standard laparoscopy. In our study, complication rate was acceptable (20%) and similar to that found during the student’s initial laparoscopic experience (data not shown). Both kidney lacerations required no treatment. One small renal vein lesion required an extra 5mm port to facilitate immediate clamping with subsequent suturing of the vessel. Other vascular lesions were small and successfully controlled without suturing.

Suturing was clearly the most demanding task using single-port access and total time noted for all students to complete this task was by far the longest of all steps during the procedure. The introduction of newer flexible and pre-bent graspers will allow better intra-abdominal mobilization of the instruments (11). Stolzenburg and colleagues evaluated pre-bent single-site instruments and verified that time required to perform pedicle dissection was significantly lower in comparison with the results of other studies (9).

Our study has some limitations. An important drawback is the lack of a control group with standard laparoscopy. Nevertheless, the aim of our study was not to compare single-port nephrectomy to standard laparoscopy, but to analyze single-port feasibility in the hands of novice surgeons. In addition, all students were already proficient in porcine laparoscopic nephrectomy and therefore their known expertise would limit such comparison. Also, sample-size is limited, especially because of the course costs and time availability of the post-graduate students. Finally, the present study was based only on a porcine model, in which nephrectomy is known to be less complex than in humans. Thus, the SILS Port® and other single port systems should be further evaluated in clinical setting before solid conclusions are drawn on its efficacy for human surgeries under inexperienced hands. Although feasible in novice hands, only experienced laparoscopic surgeons should perform LESS. In addition, the technique is favored mainly in cases where cosmesis is of paramount importance (12).

## CONCLUSIONS

To conclude, laparoscopic single-port nephrectomy using SILS^TM^ in the swine model is a feasible but relatively difficult procedure for those with intermediate laparoscopic experience. It is potentially associated with significant intraoperative complications, which may be successfully treated with the single-port system. Training aids reducing surgical time ultimately improves outcomes.
